# Early child development in children who are HIV‐exposed uninfected compared to children who are HIV‐unexposed: observational sub‐study of a cluster‐randomized trial in rural Zimbabwe

**DOI:** 10.1002/jia2.25456

**Published:** 2020-05-09

**Authors:** Robert Ntozini, Jaya Chandna, Ceri Evans, Bernard Chasekwa, Florence D Majo, Gwendoline Kandawasvika, Naume V Tavengwa, Batsirai Mutasa, Kuda Mutasa, Lawrence H Moulton, Jean H Humphrey, Melissa J Gladstone, Andrew J Prendergast, the SHINE Trial Team

**Affiliations:** ^1^ Zvitambo Institute for Maternal and Child Health Research Harare Zimbabwe; ^2^ University of Liverpool Liverpool United Kingdom; ^3^ Blizard Institute Queen Mary University of London London United Kingdom; ^4^ University of Zimbabwe Harare Zimbabwe; ^5^ Department of International Health Johns Hopkins Bloomberg School of Public Health Baltimore MD USA

**Keywords:** child development, language, motor, self‐control, HIV‐exposed uninfected, Zimbabwe

## Abstract

**Introduction:**

Exposure to maternal HIV may affect early child development (ECD), although previous studies have reported heterogeneous findings. We evaluated ECD among children who were HIV‐exposed uninfected (CHEU) and children who were HIV‐unexposed (CHU) recruited to the SHINE trial in rural Zimbabwe.

**Methods:**

SHINE was a community‐based cluster‐randomized trial of improved infant feeding and/or improved water, sanitation and hygiene. Pregnant women were enrolled between 2012 and 2015. We assessed ECD in a sub‐study at 24 months of age, between 2016 and 2017, using the Malawi Developmental Assessment Tool (MDAT; assessing motor, cognitive, language and social development); MacArthur‐Bates Communicative Development Inventory (CDI) (assessing vocabulary and grammar); A‐not‐B test (assessing object permanence); and a self‐control task. Mothers and infants were tested longitudinally for HIV. We used generalized estimating equations to compare ECD scores between CHEU and CHU, accounting for the cluster‐randomized design. Primary results were adjusted for trial‐related factors that could affect measurement reliability of ECD: study nurse, age of child, calendar month of birth, sex and randomized arm.

**Results:**

A total of 205 CHEU and 1175 CHU were evaluated. Mean total MDAT score was 90.6 (SD 8.7) in CHEU compared to 92.4 (9.1) in CHU (adjusted mean difference −1.3, 95% CI: −2.3, −0.3), driven mostly by differences in gross motor (−0.5, 95% CI: −0.9, −0.2) and language scores (−0.6, 95% CI: −1.1, −0.1). There was evidence that fine motor scores were lower in CHEU (adjusted mean difference −0.4, 95% CI: −0.8, 0.0) but no evidence of a difference in social scores (0.1, 95% CI: −0.2, 0.4). Mean MacArthur‐Bates CDI vocabulary score was 57.9 (SD 19.2) in CHEU compared to 61.3 (18.8) in CHU (adjusted mean difference −2.9 words, 95% CI: −5.7, −0.1). Object permanence and self‐control scores were similar between groups.

**Conclusions:**

CHEU in rural Zimbabwe had total child development and vocabulary scores that were approximately 0.15 standard deviations lower than CHU at two years of age. More detailed and specific studies are now needed to unravel the reasons for developmental delay in CHEU and the likelihood that these delays persist in the longer term.

## 1 Introduction

The increasing coverage of prevention of mother‐to‐child transmission (PMTCT) interventions in sub‐Saharan Africa has dramatically reduced the number of children with HIV infection. However, this success has created a growing population of HIV‐exposed but uninfected (CHEU) children, estimated to have reached 14.8 million in 2018 1, 2. CHEU have more morbidity and growth failure than children who are HIV‐unexposed (CHU) 3 and may be at increased risk of impaired neurodevelopment; however, studies comparing developmental outcomes between CHEU and CHU have heterogeneous findings. A recent systematic review, which included 11 studies in meta‐analysis, concluded that CHEU had poorer motor and cognitive development than CHU, both with and without antiretroviral therapy (ART) exposure 4. However, all six studies from outside the United States were graded “low quality” due to small sample sizes and risk of confounding. Recent studies from the ART era, published since this systematic review, have generally found poorer neurodevelopment amongst CHEU compared to CHU. At 12 months of age, a study from South Africa found increased odds of cognitive and motor delay 5. At two years of age, a study from Botswana showed expressive language delay 6 and a study from South Africa found receptive and expressive language delay 7. In contrast, a study from Uganda and Malawi did not find difference in neurodevelopmental outcomes in CHEU compared to CHU aged one to five years 8. Since the vast majority of CHEU reside in sub‐Saharan Africa, where HIV exposure, undernutrition and poverty overlap and interact, further explorations of neurodevelopment among CHEU are required. We aimed to compare neurodevelopmental outcomes at two years of age between CHEU and CHU recruited to a sub‐study of the SHINE trial in rural Zimbabwe.

## 2 Methods

### 2.1 SHINE trial

The design and methods of the Sanitation Hygiene Infant Nutrition Efficacy (SHINE) trial have been previously described 9; the full protocol and statistical analysis plan are at https://osf.io/w93hy. Between 2012 and 2015, SHINE recruited pregnant women living in two rural Zimbabwean districts with 15% antenatal HIV prevalence, high PMTCT coverage, low mother‐to‐child HIV transmission and universal breastfeeding uptake. Briefly, SHINE was a 2x2 factorial cluster‐randomized trial assessing the individual and combined effects of improved infant and young child feeding (IYCF) and improved water, sanitation and hygiene (WASH) on child stunting and anaemia (ClinicalTrials.gov NCT01824940). Overall, 5280 pregnant women were recruited from 211 clusters at a median gestational age of 12.5 weeks and randomized to standard‐of‐care (SOC); IYCF (20 g small‐quantity lipid‐based nutrient supplement (SQ‐LNS) per day from six to eighteen months of age, complementary feeding counselling); WASH (ventilated improved pit latrine and 2 hand‐washing stations, monthly liquid soap and chlorine, a play‐space to separate children from livestock and to reduce geophagia, hygiene counselling); or IYCF plus WASH (all interventions). The primary findings 10 and ECD findings among a subgroup of children at 24 months 11, 12 have been reported previously, stratified by maternal HIV status 10, 11, 13.

### 2.2 Data collection

To assess maternal and household characteristics, 11 research nurses made home visits during pregnancy at baseline (around two weeks after consent) and at 32 gestational weeks, to assess maternal and household characteristics. At baseline, maternal anthropometry and haemoglobin (Hemocue, Ängelholm, Sweden) were measured, and food insecurity, household wealth and maternal capabilities were assessed as described previously 14, 15. Infant birth date, weight and delivery details were transcribed from health facility records.

### 2.3 HIV testing

Mothers were tested during pregnancy using a rapid test algorithm (Alere Determine HIV‐1/2 test, followed by INSTI HIV‐1/2 test if positive) and offered further testing at 18 months postpartum. CHU were defined as those born to mothers testing HIV negative during pregnancy. Children who were HIV‐exposed were defined as those born to mothers testing HIV positive during pregnancy. CHEU were defined as children who were HIV‐exposed and confirmed HIV negative through 18 months of age (trial endpoint) 10. Child HIV status was determined by dried blood‐spot DNA polymerase chain reaction (PCR), plasma RNA PCR, or rapid test algorithm, depending on child age and sample type.

### 2.4 Early child development sub‐study

A sub‐study of the SHINE trial evaluated the impact of IYCF and WASH on early child development (ECD) at 24 months of age. Children who completed the 18‐month visit and turned two years (allowable age window 102 to 112 weeks) between 1 March 2016 and 30 April 2017 were eligible for the ECD sub‐study. Research nurses, who underwent three weeks of residential training and regular standardization, undertook ECD assessments using the following tools (also see Appendix S1):

1. Malawi Developmental Assessment Tool (MDAT), which measures child development in four domains (gross motor coordination, fine motor coordination, language, social). Fine motor, language and social domains also measure components of cognitive development 16.
2. MacArthur Bates Communicative Development Inventory (CDI) 17, a specific assessment of child language according to maternal report, which includes a vocabulary and grammar checklist. The test was formally adapted for Shona speakers using a rigorous method to produce a detailed protocol approved by the CDI team 18, 19.
3. A‐not‐B test, which assesses object permanence and cognition 20. This task requires the child to watch as a treat is hidden under one of two bowls; after a brief delay, the child is asked to find the treat. After two successful retrievals the object is hidden under the other bowl. The exercise is repeated ten times. Children not completing all ten tests were excluded from analyses.
4. Self‐control task 21, which assesses impulsivity. The child is required to watch as a treat is promised to them, but they have to wait for two minutes to take it. The test is first conducted with a covered treat (hidden), then an uncovered treat (not hidden). Self‐control was defined as a child who waited for two minutes.

Children who scored “moderate to severe” on the Washington screen for disability 22 were assessed and referred for appropriate services, but were excluded from analyses. Children of HIV‐positive mothers who could not be confirmed HIV negative at 18 months of age were also excluded from analyses.

### 2.5 Statistical analyses

The purpose of this analysis was to estimate the differences in ECD outcomes at 24 months of age between CHEU and CHU. We have previously reported the effects of the randomized SHINE interventions, which differed between these two groups of children 11, 12. Among CHU, there was little evidence of effect of either intervention on any measure of child development; by contrast, among CHEU, there were large benefits of the combined IYCF plus WASH intervention on MDAT and MacArthur CDI vocabulary scores, but no evidence of effects from either the IYCF or WASH intervention when implemented alone. Accordingly, before comparing ECD measures between CHEU and CHU, we first tested for interactions between HIV exposure status and each intervention arm for the total MDAT score and for the MacArthur Bates vocabulary scores; these were considered important if the interaction term was significant (*p* < 0.05, Wald test), or had a sizeable point estimate (difference‐of‐differences >0.25 SD). For both outcomes there were important interactions between HIV exposure status and IYCF plus WASH but not for the IYCF or WASH intervention alone (IYCF plus WASH *p* = 0.01 for MDAT, *p* = 0.02 for MacArthur Bates; IYCF alone *p* = 0.63 and *p* = 0.68 respectively; and WASH alone *p* = 0.91 and *p* = 0.64 respectively). Therefore, to ensure an unbiased estimate of the difference in ECD associated with HIV exposure, we excluded children randomized to the IYCF plus WASH arm from the main analysis.

In a sensitivity analysis, we restricted analyses to CHEU versus CHU in the SOC trial arm only (i.e. among children who had received no trial interventions). In another sensitivity analysis, we excluded children whose mothers were HIV negative during pregnancy but seroconverted to HIV by 18 months postpartum, as these children would have been exposed to HIV during breastfeeding. A subgroup analysis by child sex was planned if there was a significant interaction between sex and HIV exposure; these were considered important if the interaction term was significant (*p* < 0.05, Wald test), or had a sizeable point estimate (RR > 2 or <0.5 when comparing either of the two ratio‐of‐ratios for dichotomous outcomes, or difference‐of‐differences >0.25 SD when comparing continuous outcomes).

We compared baseline characteristics between groups while handling within‐cluster correlation using multinomial and ordinal regression models with robust variance estimation, and Somers’ D for medians. To compare ECD outcomes between CHEU and CHU, we used generalized estimating equations (GEE) with an exchangeable working correlation structure, adjusted for trial arm only (Model 1). A log‐binomial specification was used to facilitate the estimation of relative risks (RR). We then undertook an adjusted analysis that included trial factors which may have biased ECD measurement (study nurse carrying out the assessment, exact child age at assessment, calendar month of birth), in addition to trial arm (Model 2). This analysis estimates ECD differences between CHEU and CHU which may be a result of any factor (biologic, socioeconomic or demographic) and represents the public health impact of exposure to maternal HIV infection on ECD. This estimate may be useful in planning intervention programmes to improve ECD in HIV‐affected populations. Finally, we undertook a third analysis adjusting for potential socioeconomic and demographic confounders, defined as baseline factors which were univariably associated with HIV exposure (*p* < 0.05), univariably associated with the ECD outcome, and not likely to be on the causal pathway leading from HIV exposure to neurodevelopmental outcomes (Model 3). For example, we did not adjust for birth weight because it is likely to be on the causal pathway. This analysis estimates the biologic impact on ECD outcomes of exposure to maternal HIV infection. All analyses were carried out using Stata versions 14.1 and 15.1.

### 2.6 Ethics

Mothers provided written informed consent for the main trial and ECD sub‐study. The Medical Research Council of Zimbabwe and the Institutional Review Board of the Johns Hopkins Bloomberg School of Public Health approved the study protocol. The trial was registered at ClinicalTrials.gov (NCT01824940) and all study tools are available at https://osf.io/w93hy/.

## 3 Results

**Figure 1:**
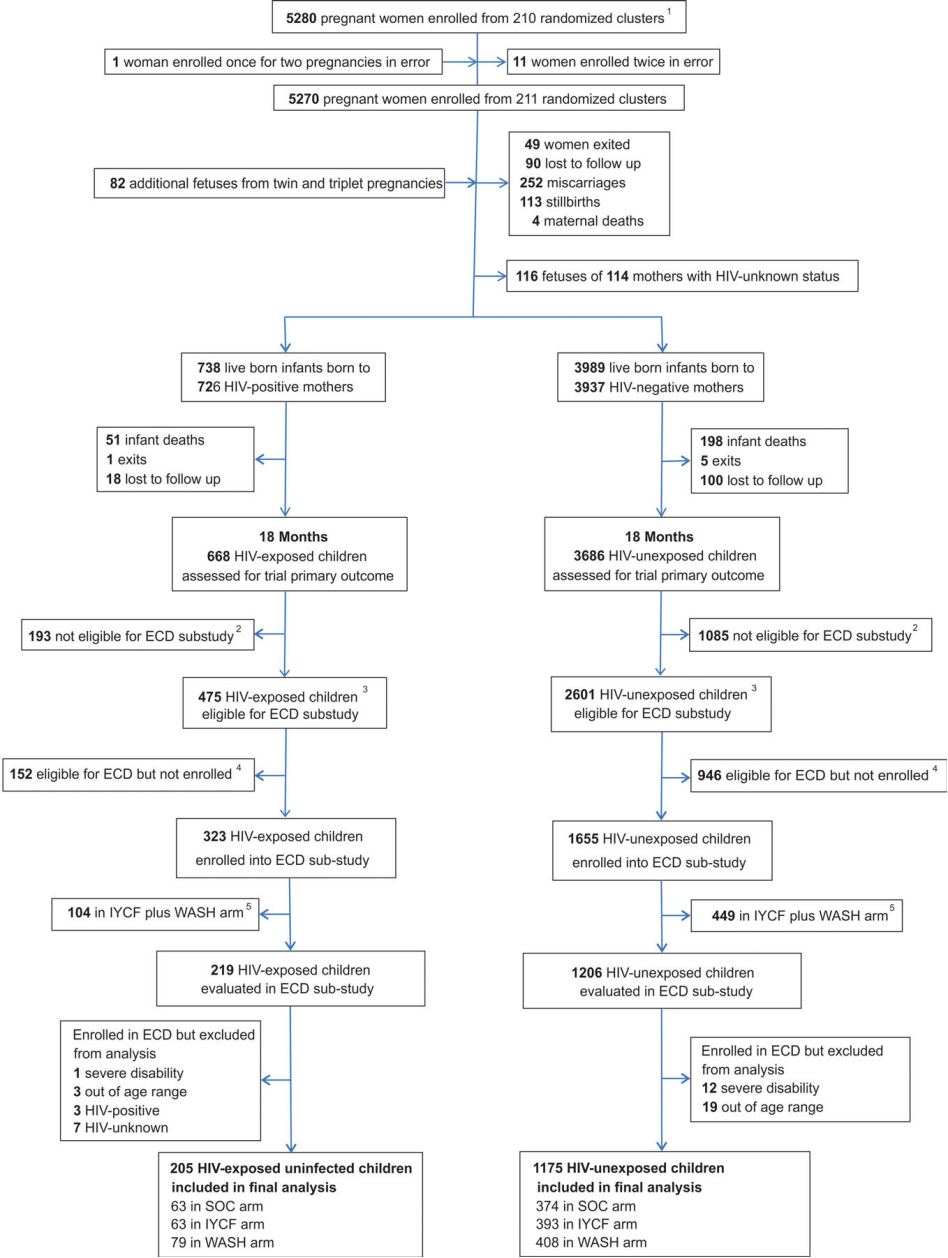
Flow of participants through the SHINE early child development (ECD) sub‐study. ^1^In all, 212 clusters were randomized, 53 in each of the four trial arms. After randomization, 1 cluster was excluded as it was determined to be in an urban area, 1 cluster was excluded as the village health worker covering it mainly had clients outside the study area, and 1 more was merged into a neighbouring cluster based on subsequent data on village health worker coverage. Three new cluster designations were created due to anomalies in the original mapping: for 2 of these, the trial arm was clear; the third contained areas that were in 2 trial arms, and was assigned to the underrepresented arm, resulting in 53 clusters in each arm. All of this occurred before enrolment began. When enrolment was completed, however, there was 1 standard‐of‐care cluster in which no women were enrolled, leaving a total of 211 clusters available for analysis. ^2^Children were not eligible for the ECD sub‐study if they turned two years of age (allowable range 102 to 112 weeks) before 1 March 2016. ^3^Children were eligible for the ECD sub‐study if they turned two years of age (allowable range 102 to 112 weeks) between 1 March 2016, and April 30, 2017. ^4^Children were eligible for the ECD sub‐study because they turned two years of age (allowable range 102 to 112 weeks) between 1 March 2016, and 30 April 2017, but they were not contactable or were not approached for consent because the number of children becoming 102 to 112 weeks of age between 1 March 2016, and 30 April 2017, exceeded the capacity of the 11 ECD‐trained nurses. ^5^Children in the WASH plus IYCF combined arm were excluded from this analysis because of an interaction between HIV exposure and IYCF plus WASH for the total MDAT score (*p* = 0.008, Wald test). IYCF: infant and young child feeding; SOC: standard of care; WASH: water, sanitation and hygiene.

Among 5280 enrolled pregnant women, there were 738 HIV‐exposed and 3989 HIV‐unexposed live births; of these, 323 HIV‐exposed and 1655 CHU were assessed in the ECD sub‐study at two years of age (Figure 1). Mothers of children included in the ECD sub‐study were about two years older compared to mothers of those not included; other baseline characteristics were broadly similar (as reported previously 11, 12 and shown in Table S1).

Of the 323 HIV‐exposed children evaluated, 104 (32%) were randomized to the IYCF plus WASH arm and were excluded from analysis. Among the 219 remaining CHEU, one child (0.5%) was excluded due to severe disability, and three (1.4%) were excluded because they were subsequently found to be outside the pre‐defined age window (102 to 112 weeks). Three of the 219 HIV‐exposed children (1%) were HIV positive and 7 (4%) were HIV‐unknown; these 14 children were excluded from the analysis, leaving a total of 205 CHEU. Of the 1655 CHU evaluated, 449 (27%) randomized to the IYCF plus WASH arm were excluded. Among the 1206 remaining children, 12 (1%) were excluded due to severe disability, and 19 (2%) were excluded because they were subsequently found to be outside the pre‐defined age window. Overall, 205 CHEU and 1175 CHU were therefore included in this analysis (Figure 1).

### 3.1 Baseline characteristics

Baseline characteristics of mothers, households and children in this analysis are shown in Table 1. HIV‐positive mothers were on average four years older, had less education and a higher parity than HIV‐negative mothers. HIV‐positive mothers also had significantly more depressive symptoms and felt greater time stress compared to HIV‐negative mothers. However, HIV‐positive mothers had greater confidence in their parenting skills, as assessed by the maternal self‐efficacy questions. Households of HIV‐positive women tended to be in lower wealth quintiles and have greater food insecurity than households of HIV‐negative women. Mean birth weight was lower in CHEU compared to CHU. Other baseline characteristics were similar between HIV positive and HIV‐negative mothers and their children. Among the 216 HIV‐positive mothers, 185 (86%) had documented exposure to ART during pregnancy. Of these 185 ART‐exposed women, almost three quarters were on a tenofovir‐based regimen. Only one HIV‐positive mother was taking a protease inhibitor (atazanavir). Mean (SD) pregnancy CD4 count was 456 (210) cells/μL (Table 1).

**Table 1:** Maternal, household and child baseline characteristics among CHEU and CHU

Baseline characteristic^a^	CHEU	CHU	*p* value
Mothers [N]	216	1195	
Children [N]	219	1206	
Trial arm			
SOC	68/219 (31.1%)	387/1206 (32.1%)	0.77
IYCF	68/219 (31.1%)	398/1206 (33.0%)	
WASH	83/219 (37.9%)	421/1206 (34.9%)	
Maternal characteristics			
Mean age (SD), years	30.7 (6.3)	26.4 (6.6)	<0.0001
Mean height (SD), cm	160.6 (6.5)	160.3 (6.0)	0.50
Mean MUAC (SD), cm	26.5 (3.2)	26.5 (3.2)	0.92
Mean completed schooling (SD), years	9.1 (2.1)	9.6 (1.8)	0.0008
Median parity (IQR)	2.5 (2, 3)	2 (1, 3)	<0.0001
Married	191/200 (95.5%)	1073/1125 (95.4%)	0.94
Employed	18/208 (8.7%)	107/1110 (9.6%)	0.66
Religion			
Apostolic	100/216 (46.3%)	555/1195 (46.4%)	0.057
Other Christian religions	80/216 (37.0%)	509/1195 (42.6%)	
Other non‐Christian religions	36/216 (16.7%)	131/1195 (11.0%)	
Maternal capabilities^b^			
Mean gender norms and attitudes score (SD)	2.32 (0.82)	2.30 (0.82)	0.94
Mean perceived social support score (SD)	3.54 (0.64)	3.61 (0.58)	0.15
Mean perceived physical health (SD)	3.54 (1.00)	3.42 (1.00)	0.14
Mean mothering self‐efficacy score (SD)	4.05 (0.37)	3.98 (0.40)	0.020
Mean perceived time stress score (SD)	2.82 (0.75)	2.64 (0.70)	0.012
Median decision‐making autonomy (IQR)	5 (4,5)	5 (4,5)	0.78
Mean Edinburgh postnatal depression score (SD)	3.31 (4.59)	2.47 (3.78)	0.006
HIV disease severity and treatment			
Mean CD4 count in pregnancy (SD), cells/μL^c^	456 (210)	N/A	
Documented antiretroviral therapy during pregnancy^d^	185/216 (85.7%)	N/A	
Tenofovir disoproxil fumarate‐based ART regimen	134/185 (72.4%)	N/A	
Zidovudine‐based ART regimen	29/185 (15.7%)	N/A	
Other/unknown ART regimen^e^	22/185 (11.9%)	N/A	
Documented co‐trimoxazole prophylaxis during pregnancy^f^	137/216 (63.4%)	N/A	
Household characteristics			
Median household size (IQR)	4 (3, 6)	5 (3, 6)	0.32
Median coping strategies index score (IQR)^g^	2 (0, 11)	1 (0, 8)	0.006
Wealth quintile^h^			
Lowest	50/209 (23.9%)	193/1115 (17.3%)	0.091
Second	43/209 (20.6%)	218/1115 (19.6%)	
Middle	45/209 (21.5%)	233/1115 (20.9%)	
Fourth	36/209 (17.2%)	244/1115 (21.9%)	
Highest	35/209 (16.8%)	227/1115 (20.4%)	
Child characteristics			
Female	105/219 (48.0%)	609/1206 (50.5%)	0.48
Mean birth weight (SD), kg	3.00 (0.49)	3.09 (0.47)	0.057
Birth weight <2500 g	24/219 (11.4%)	100/1117 (8.7%)	0.39
Institutional delivery	183/212 (86.3%)	1015/1139 (89.1%)	0.23
Vaginal delivery	198/214 (92.8%)	1086/1169 (92.9%)	0.84

CHEU, children HIV‐exposed but uninfected; CHU, children HIV‐unexposed; IQR, interquartile range; IYCF, Infant and Young Child Feeding; MUAC, Mid‐upper arm circumference; SD, standard deviation; SOC, Standard of Care; WASH, Water and Sanitation/Hygiene.

a Baseline variables presented for mothers who had live births. Maternal and household data were collected about two weeks after consent (approximately 14 weeks gestation); this gap created opportunity for loss to follow‐up between consent and baseline, thus the number of mothers completing baseline visit is smaller than the number of mothers with live births. Baseline for infants was at birth. Values are %, unless noted. For variables where [n] is not stated, <3% of data are missing based on number of baseline visits completed

b maternal capabilities scores are described in Matare et al. 15 whereby scores generated for each of the caregiver capabilities measure, higher values represent greater decision‐making autonomy, more liberal gender norm attitudes, higher levels of depressive symptoms, greater mothering self‐efficacy, perceptions of better physical health, perceptions of more social support and perceptions of high levels of time stress

c CD4 count at baseline visit, or at 32 gestational week visit if no baseline result

d documented antiretroviral therapy use during pregnancy; only available for 187/216 (85.7%) women;

e includes non‐TDF‐ or AZT‐based regimens; use of both TDF and AZT during pregnancy (including switching regimens); or undocumented antiretroviral therapy regimen

f documented co‐trimoxazole prophylaxis use during pregnancy; only available for 134/216 (63.4%) women

g the Coping Strategy Index is a measure of household food security (CARE and World Food Program, 2003).

h described in Chasekwa et al. 14.

### 3.2 ECD outcomes

ECD outcomes in CHEU and CHU are shown in Table 2. The mean difference in scores between CHEU and CHU are presented adjusted for trial arm only (Model 1); adjusted for trial arm plus other trial factors which may have biased ECD measurements (Model 2); and adjusted for trial arm, trial factors and potential baseline confounders (Model 3). Mean (SD) total MDAT score was 90.6 (8.7) in CHEU compared to 92.4 (9.1) in CHU. The adjusted mean difference (95% CI) was −1.3 (−2.3, −0.3), in Model 2; and −1.1 (−2.1, 0.0) in Model 3. These differences are equivalent to a difference of 0.15 and 0.13 SD respectively in total MDAT score between groups. Within the MDAT assessment, there was greatest evidence for differences in gross motor scores (adjusted mean difference −0.5 (−0.9, −0.2) in both Model 2 and Model 3); and language scores (−0.6 (−1.1, −0.1) in Model 2 and −0.5 (−1.0, 0.0) in Model 3). Both models suggested there was some evidence that fine motor scores were lower in CHEU but no evidence that social scores differed between CHEU and CHU.

**Table 2:** ECD outcomes among CHEU and CHU at two years of age

Continuous ECD outcomes	CHEU	CHU	Mean difference (95% CI)		
	Mean (SD) N = 205	Mean (SD) N = 1175	Model 1^a^	Model 2^b^	Model 3^c^
Malawi development assessment tool					
Total score	90.6 (8.7)	92.4 (9.1)	−1.6 (−2.7, −0.5)	−1.3 (−2.3, −0.3)	−1.1 (−2.1, 0.0)
Gross motor	23.0 (2.9)	23.7 (3.1)	−0.6 (−0.9, −0.3)	−0.5 (−0.9, −0.2)	−0.5 (−0.9 −0.2)
Fine motor	22.8 (2.9)	23.2 (2.5)	−0.4 (−0.8, 0.0)	−0.4 (−0.8, 0.0)	−0.3 (−0.7, 0.1)
Language	20.5 (3.9)	21.4 (4.2)	−0.7 (−1.3, −0.2)	−0.6 (−1.1, −0.1)	−0.5 (−1.0, 0.0)
Social	24.3 (2.3)	24.2 (2.3)	0.1 (−0.2, 0.4)	0.1 (−0.2, 0.4)	0.2 (−0.1, 0.5)
MacArthur‐Bates CDI vocabulary checklist	57.9 (19.2)	61.3 (18.8)	−3.3 (−6.1, −0.4)	−2.9 (−5.7, −0.1)	−3.5 (−6.3, −0.8)
Object permanence (A‐not‐B test)	7.8 (1.4)	7.8 (1.4)	0.0 (−0.2, 0.2)	0.0 (−0.2, 0.2)	0.0 (−0.2, 0.2)

Dichotomous ECD outcomes	n/N (%)	n/N (%)	Relative risk (95% CI)		
			Model 1^a^	Model 2^b^	Model 3^c^
Self‐control task					
Hidden	129/200 (65%)	737/1151 (64%)	1.01 (0.90, 1.13)	1.00 (0.90, 1.13)	1.01 (0.80, 1.21)
Not hidden	90/198 (46%)	518/1141 (45%)	1.00 (0.85, 1.17)	0.99 (0.85, 1.16)	1.01 (0.87, 1.13)
MacArthur‐Bates CDI grammar checklist					
Uses plurals	37/205 (18%)	265/1175 (23%)	0.85 (0.65, 1.12)	0.89 (0.68, 1.17)	0.82 (0.63, 1.06)
Uses imperatives/progressives	147/205 (72%)	850/1175 (72%)	0.99 (0.92, 1.07)	1.01 (0.94, 1.09)	0.99 (0.92 1.07)
Combines two words	200/205 (98%)	1160/1175 (99%)	0.99 (0.97, 1.01)	0.99 (0.97, 1.01)	0.99 (0.97, 1.01)

In this analysis, children in the combined IYCF plus WASH arm were removed from analyses, as explained in the Methods section, due to an interaction between HIV exposure status and the IYCF plus WASH arm, for the total MDAT and MacArthur Bates CDI tests; there were no interactions between HIV exposure status and other trial arms for these outcomes. CDI, communicative development inventory; CHEU, children HIV‐exposed but uninfected; CHU, children HIV‐unexposed; CI, confidence interval; ECD, early child development.

a Model 1: regression models adjusted for trial arms only

b Model 2: regression models adjusted for factors that could affect measurement reliability of early child development: study nurse, calendar age of child at assessment, sex, and calendar month of birth, in addition to trial arms

c Model 3: regression models adjusted for study nurse, calendar age of child at assessment, sex, and calendar month of birth and trial arms, in addition to baseline covariates that were associated with the exposure (HIV exposure status) and outcome on univariable analysis. The following covariates were offered into models: maternal age, height, parity, religion, mid‐upper arm circumference (MUAC), education, marital status and employment status, and household wealth and size. The variables retained for each outcome were as follows. Malawi Development Assessment Tool: study nurse, calendar age of child at assessment, sex, and calendar month of birth, trial arms, maternal education, household wealth. MacArthur‐Bates CDI vocabulary and grammar checklists: study nurse, calendar age of child at assessment, sex, and calendar month of birth, trial arms, maternal age, parity, education and household wealth. A‐not‐B test: study nurse, calendar age of child at assessment, sex, and calendar month of birth, trial arms, household wealth. Self‐control task: study nurse, calendar age of child at assessment, sex, and calendar month of birth, trial arms, maternal education.

The mean number of reported words used by children in the MacArthur‐Bates CDI was 57.9 (SD 19.2) for CHEU versus 61.3 (18.8) for CHU. The adjusted mean difference (95% CI) was −2.9 (−5.7, −0.1) in Model 2, and −3.5 (−6.3, −0.8) in Model 3**.** These differences are equivalent to a difference of 0.15 and 0.18 SD respectively in MacArthur Bates CDI vocabulary scores between groups. MacArthur Bates CDI grammar scores were similar between CHEU and CHU (Table 2).

There was no evidence of differences in object permanence or self‐control measures between CHEU and CHU (Table 2).

### 3.3 Sensitivity and subgroup analyses

A sensitivity analysis, which excluded seven CHU whose mothers seroconverted to HIV by 18 months postpartum, showed very similar findings (data not shown). When analyses were restricted only to children randomized to the SOC arm (i.e. removing children who received any IYCF or WASH interventions), overall findings were similar, albeit with less precise estimates (Table 3).

**Table 3:** Early child development outcomes among CHEU and CHU at two years of age in the standard of care arm

Continuous ECD Outcomes	CHEU^a^	CHU^a^	Mean difference (95% CI)
	Mean (SD) N = 63	Mean (SD) N = 373	
Malawi development assessment tool			
Total score	90.7 (8.1)	92.7 (9.5)	−1.8 (−3.7, 0.1)
Gross motor	23.0 (2.6)	23.8 (3.3)	−0.8 (−1.5, −0.1)
Fine motor	22.9 (2.4)	23.4 (2.7)	−0.4 (−1.0, 0.1)
Language	20.7 (3.8)	21.4 (4.2)	−0.6 (−1.7, 0.4)
Social	24.1 (2.1)	24.2 (2.1)	0.0 (−0.5, 0.4)
MacArthur‐Bates CDI vocabulary checklist	56.9 (18.3)	61.3 (18.7)	−4.2 (−8.3, −0.2)
A‐not‐B test	7.8 (1.3)	7.8 (1.3)	0.0 (−0.4, 0.4)

Dichotomous ECD Outcomes	n/N (%)	n/N (%)	Unadjusted relative risk^a^ (95% CI)
Self‐control task			
Hidden	43/59 (73%)	237/365 (65%)	1.13 (0.96, 1.34)
Not hidden	31/58 (54%)	170/359 (47%)	1.13 (0.87, 1.47)
MacArthur‐Bates CDI grammar checklist			
Uses plurals	8/63 (13%)	61/373 (16%)	0.82 (0.47, 1.44)
Uses imperatives/progressives	47/63 (75%)	284/373 (76%)	0.98 (0.86, 1.12)
Combines two words	61/63 (97%)	369/373 (99%)	0.98 (0.94, 1.02)

CDI, communicative development inventory.

a In this analysis, children in the IYCF, WASH and combined IYCF + WASH arms were removed from analyses, meaning only children in the standard‐of‐care arm, who received no exposure to trial interventions, are included here.

Child sex did not modify the effects of HIV exposure on any ECD outcome (MDAT total score difference‐in‐difference −1.41 95% CI: −3.99, 1.18; *p* = 0.29, and MacArthur‐Bates CDI score difference‐in‐difference −0.97 95% CI: −7.10, 5.16; *p* = 0.76).

## 4 Discussion

There is an expanding global population of children who are HIV‐exposed but uninfected 1. Concerns regarding neurodevelopmental impairment in CHEU have been raised 4, 23, but there is a paucity of robust data. We evaluated ECD among a large group of children recruited to the SHINE trial in rural Zimbabwe, using well‐validated direct and parental report assessments across a range of ECD domains. Overall, we found evidence of differences in motor and language outcomes between CHEU and CHU, with the MDAT score (a measure of overall child development) and MacArthur Bates CDI vocabulary score equivalent to about 0.15 standard deviations lower in CHEU. Our study clarifies a current concern in this PMTCT era – that there is a definitive delay in motor and language acquisition within the first 1000 days among HIV‐negative children who are born to HIV‐positive mothers. Longer‐term and more detailed studies are necessary to determine whether these differences have an impact on school performance and adult human capital; there is also need to elucidate underlying causes and effective interventions.

Previous comparisons of neurodevelopment between CHEU and CHU have been heterogeneous 4. Two of the largest studies from southern Africa used the Bayley Scales of Infant and Toddler Development and had conflicting findings. In Botswana, between 2010 and 2012 when only one‐third of mothers received combination ART during pregnancy and <10% of CHEU were breastfed compared to >99% of CHU, there was little evidence of differences in neurodevelopment at 24 months of age (N = 724) 6. However, in the era of combination maternal ART and breastfeeding in South Africa, CHEU had higher odds of cognitive and motor, although not language, delay at 13 months of age (N = 521) 5. Our study population and findings are closer to the South African study; however, in using more detailed language assessments for two‐year‐old children, we additionally found differences in vocabulary scores between groups. CHEU knew on average three to four fewer words than CHU, which is a relatively large difference at this age. Since language is arguably the most sensitive indicator of child development at two years of age 24, 25, this could translate into important differences in school attainment at older ages.

We tested children using age‐appropriate HIV tests, enabling us to exclude children living with HIV and children with unknown HIV status from the analyses, and to conduct a sensitivity analysis excluding children whose mothers seroconverted to HIV during the breastfeeding period. We used several different developmental assessment tools across a broad range of domains, and specifically adapted them for use in rural Zimbabwe (for example, all toys were bought locally and therefore likely to be familiar to children). The MacArthur Bates CDI is a detailed assessment of language, including both vocabulary and grammar, and we adapted and validated 11 the assessment for use among Shona‐speaking households. We also undertook rigorous quality control throughout the study period, to optimize reliability. Previous studies in high‐income settings have often been limited by incomparable control groups (e.g. more maternal recreational drug use and less breastfeeding among HIV‐affected families), and may not be representative of HIV‐exposed children in low‐ and middle‐income countries 4, 23. Previous studies in sub‐Saharan Africa have been limited by small sample sizes, differences in breastfeeding between HIV‐exposed and unexposed infants, and developmental assessment tools which may not have been culturally adapted 4, 23. In SHINE, 15% of mothers were HIV‐positive at enrolment, the majority were taking ART for PMTCT and HIV transmission was low, allowing a relatively large comparison of CHEU and CHU; our sample of 1380 children makes this the largest study to date. Breastfeeding was universal in this population and exclusive breastfeeding rates in the first six months after birth were high 26.

Here we offer three potential explanations for the reduction in ECD scores seen among CHEU. First, there may be social differences between HIV‐affected and HIV‐unaffected families, including disparities in household wealth and maternal education. In SHINE, compared to HIV‐negative mothers, HIV‐positive mothers were poorer and had completed slightly less schooling; both poverty and maternal education are strongly associated with ECD 27. However, estimated mean differences in ECD scores between groups were similar in Model 3 (after adjusting for potential social confounders) compared to Model 2 (which adjusted only for trial factors) suggesting that most of the adverse effect on ECD outcomes associated with maternal HIV were likely biologic rather than social, although we cannot exclude residual confounding due to unmeasured social differences. Second, maternal HIV may affect care‐taking capabilities. In Uganda, the observed quality of the caregiving environment was lower among HIV‐affected families, and was associated with reduced language scores 28. In SHINE, compared to HIV‐negative women, those who were HIV‐positive had more depression, a well‐documented determinant of poor infant neurodevelopmental outcomes 29 which may have mediated the effect of maternal HIV infection on ECD outcomes. However, notably, a caregiver training intervention did not improve neurodevelopment among CHEU in a cluster‐randomized trial in Uganda, despite improving the quality of caregiving 30. Third, HIV‐specific biomedical factors may contribute to neurodevelopmental impairment, including effects of the virus itself, immunosuppression, co‐infections (including CMV 31), and ART. It is plausible that direct exposure to maternal HIV virions *in utero* can affect brain growth during a vulnerable period of development 23. A recent study from South Africa demonstrated a relationship between cumulative maternal HIV viraemia during pregnancy (expressed as viraemia copy‐years) and higher odds of motor and expressive language delay 32. Studies from South Africa also showed that CHEU had white matter abnormalities as early as two to four weeks of age 33 and as late as seven years of age 34. Several animal and human studies have identified a pivotal role for immune cells in brain development 35; maternal HIV may indirectly affect foetal neurodevelopment through the impact of maternal immunosuppression during pregnancy on foetal immune ontogeny 3, 36. Characterizing immune development, integrity of the blood–brain barrier in early life, and the role of neuro‐inflammation will be necessary to fully understand and address the drivers of cognitive delays in CHEU.

It is critical to evaluate whether interventions can reduce the neurodevelopmental gap between CHEU and CHU. We showed previously in SHINE that CHEU randomized to the combined IYCF plus WASH intervention had significant improvements in ECD scores 12. This is in contrast to CHU, who had no meaningful improvements in ECD scores in response to the IYCF or WASH interventions, whether delivered alone or in combination. CHEU may be particularly responsive to targeted interventions, although we lack understanding of why CHEU responded to the combined intervention, but showed no evidence of improved ECD with either the IYCF or WASH intervention alone.

This analysis has some important limitations. First, not all children recruited to SHINE were eligible to join the ECD sub‐study, and among those eligible not all children were enrolled. It is possible selection may have biased our comparison between CHEU and CHU, although baseline characteristics among those not eligible and eligible but not enrolled were similar to those enrolled into the ECD sub‐study. Second, because CHEU and CHU responded differently to the trial interventions, we excluded all children randomized to the IYCF plus WASH arm after interaction testing, which reduced the numbers in this analysis. Third, we did not explore the relationship between ART use or regimen and ECD. Most HIV‐positive mothers in SHINE received ART during the antenatal period, three‐quarters of whom were on a tenofovir‐based regimen. Although trial staff promoted uptake of PMTCT at local clinics, we did not provide HIV care or dispense ART. There were too few ART‐unexposed children (N = 31) to enable a meaningful analysis of the impact of ART (or specific regimens) on neurodevelopment. Furthermore, mothers were not randomized to ART use or regimen, so it would be difficult to ascertain whether any associations were due to ART or confounding. A recent sub‐analysis from the PROMISE trial, in which mothers were randomized to ART regimens, found that triple antiretroviral use during pregnancy and breastfeeding did not influence neurodevelopmental outcomes through five years of age among CHEU 8.

This study adds to the growing literature on neurodevelopment in CHEU and CHU. In summary, we show that motor and language skills at two years of age were lower among CHEU compared to CHU. These differences may have substantial impact at a population level in areas of high antenatal HIV prevalence. Longer‐term studies are needed to evaluate whether these differences in motor and language outcomes at age two years translate into meaningful differences in school attainment and adult economic productivity. There is a need to evaluate whether additional ECD‐specific interventions could further improve the long‐term developmental potential of this vulnerable group of children.

## 5 Conclusions

CHEU in rural Zimbabwe had total child development and vocabulary scores that were approximately 0.15 standard deviations lower than CHU at two years of age. More detailed and specific studies are now needed to unravel the reasons for developmental delay in CHEU and the likelihood that these delays persist in the longer term.

## Competing interests

We declare no competing interests.

## Authors’ contributions

RN, CE and JC led data analysis and interpretation of this sub‐study and wrote the first draft of the manuscript. RN developed and managed all information technology, data management, and data analysis for the SHINE trial and this sub‐study. JC, GK, MJG and AJP designed and directed implementation of the neurodevelopment sub‐study. CE, BC, LHM and JHH contributed to data analysis and interpretation of this sub‐study. JHH was the principal investigator of the SHINE trial. FDM and BM supervised all data collection. NVT managed field operations. LHM was the senior statistician. AJP directed all clinical and laboratory aspects of the trial and managed the data collection and laboratory teams. KM managed the laboratory and led HIV testing and interpretation. All authors contributed to, reviewed and approved this manuscript.

## Abbreviations

ART, antiretroviral therapy; CDI, MacArthur‐Bates Communicative Development Inventory; ECD, early child development; GEE, generalized estimating equations; CHEU, children HIV‐exposed uninfected; CHU, children HIV‐unexposed; IYCF, infant and young child feeding; MDAT, Malawi Developmental Assessment Tool; PCR, polymerase chain reaction; PMTCT, prevention of mother‐to‐child transmission; RR, relative risk; SD, standard deviation; SHINE, Sanitation Hygiene Infant Nutrition Efficacy; SOC, standard‐of‐care; SQ‐LNS, small‐quantity lipid‐based nutrient supplement; WASH, water, sanitation and hygiene.

## Funding

The SHINE trial is funded by the Bill & Melinda Gates Foundation (OPP1021542 and OPP113707); Department for International Development (DFID), UK; Wellcome Trust, UK (093768/Z/10/Z, 108065/Z/15/Z and 203905/Z/16/Z); Swiss Agency for Development and Cooperation (SDC); National Institutes of Health, USA (2R01HD060338‐06); and UNICEF (PCA‐2017‐0002). Role of funder: Study funders approved the trial design, but were not involved in data collection, analysis, or interpretation, nor decisions related to publication. The corresponding author had full access to all study data and ultimate responsibility for the decision to submit for publication.

## Supporting information


**Table S1.** Baseline characteristics of mothers and infants who were enrolled versus not enrolled into the ECD sub‐study by HIV exposure status.

**Appendix S1.** Supplementary methods.

**Appendix S2.** SHINE Team list.
