# Supplementary material for: Early child development in children who are HIV‐exposed uninfected compared to children who are HIV‐unexposed: observational sub‐study of a cluster‐randomized trial in rural Zimbabwe
Source: J Int AIDS Soc. 2020 May 9;23(5):e25456. doi: 10.1002/jia2.25456 (PMC7318086; doi:10.1002/jia2.25456)
Supplement: Supplementary file 1 — Table S1. Baseline characteristics of mothers and infants who were enrolled versus not enrolled into the ECD sub‐study by HIV exposure status. [file JIA2-23-e25456-s001.docx]

**Table S1: Baseline characteristics of mothers and infants who were enrolled versus not enrolled into the ECD sub-study by HIV exposure status**

|  | **HIV infected women and HIV-exposed children** | | |  | **HIV uninfected women and HIV-unexposed children** | | |
| --- | --- | --- | --- | --- | --- | --- | --- |
| **Baseline characteristic^1^** | **Enrolled into ECD** | **Not enrolled into ECD** | *P value* |  | **Enrolled into ECD** | **Not enrolled into ECD** | *P value* |
| Mothers, N | 318 | 408 |  |  | 1639 | 2298 |  |
| Infants, N | 323 | 415 |  |  | 1655 | 2334 |  |
| Mothers completing baseline visit | 313 | 404 |  |  | 1550 | 2139 |  |
| **Maternal characteristics** |  |  |  |  |  |  |  |
| Mean age (SD), years | 30.7 (6.1) | 28.0 (6.1) | <0.001 |  | 26.5 (6.6) | 25.0 (6.5) | <0.001 |
| Mean height (SD), cm | 160.1 (6.5) | 160.2 (6.0) | 0.89 |  | 160.3 (5.9) | 160.0 (5.8) | 0.097 |
| Mean MUAC (SD), cm | 26.5 (3.1) | 26.1 (2.8) | 0.069 |  | 26.5 (3.1) | 26.4 (3.1) | 0.23 |
| Mean completed schooling (SD), years | 9.1 (2.0) | 9.1 (2.2) | 0.91 |  | 9.6 (1.8) | 9.6 (1.8) | 0.87 |
| Median parity (IQR) | 2 (1, 3) | 2 (1, 3) | 0.002 |  | 2 (1, 3) | 2 (1, 3) | 0.005 |
| Married | 276/292 (94.5%) | 367/390 (94.1%) | 0.85 |  | 1475/1540 (95.8%) | 2071/2177 (95.1%) | 0.36 |
| Employed | 26/308 (8.4%) | 41/402 (10.2%) | 0.45 |  | 145/1539 (9.4%) | 166/2116 (7.8%) | 0.13 |
| Religion: |  |  | 0.64 |  |  |  | 0.23 |
| Apostolic | 146/318 (45.9%) | 184/408 (45.1%) |  |  | 760/1639 (46.4%) | 1002/2298 (43.6%) |  |
| Other Christian | 121/318 (38.1%) | 167/408 (40.9%) |  |  | 687/1639 (41.9%) | 998/2298 (43.4%) |  |
| Other | 51/318 (16.0%) | 57/408 (14.0%) |  |  | 192/1639 (17.7%) | 298/2298 (13.0%) |  |
| HIV disease severity and treatment: |  |  |  |  |  |  |  |
| Mean CD4 count in pregnancy (SD), cells/uL^2^ | 461 (218) | 488 (226) | 0.068 |  | N/A | N/A | N/A |
| Documented antiretroviral therapy during pregnancy^3^ | 275/280 (98.2%) | 312/321 (97.2%) | 0.41 |  | N/A | N/A | N/A |
| Documented co-trimoxazole prophylaxis during pregnancy^4^ | 200/255 (78.4%) | 202/274 (73.7%) | 0.21 |  | N/A | N/A | N/A |
| **Household characteristics** |  |  |  |  |  |  |  |
| Median household size (IQR) | 4 (3, 6) | 4 (3, 6) | 0.19 |  | 5 (3, 6) | 5 (3, 6) | 0.39 |
| Wealth Quintile^5^ |  |  | 0.32 |  |  |  | 0.13 |
| Lowest | 78/309 (25.2%) | 113/403 (28.0%) |  |  | 264/1545 (17.1%) | 414/2114 (19.6%) |  |
| Second | 61/309 (19.7%) | 104/403 (25.8%) |  |  | 307/1545 (19.9%) | 403/2114 (19.1%) |  |
| Middle | 63/309 (20.4%) | 76/403 (18.9%) |  |  | 322/1545 (20.8%) | 419/2114 (19.8%) |  |
| Fourth | 53/309 (17.2%) | 52/403 (12.9%) |  |  | 344/1545 (22.3%) | 430/2114 (20.3%) |  |
| Highest | 54/309 (17.5%) | 58/403 (14.4%) |  |  | 308/1545 (19.9%) | 448/2114 (21.2%) |  |
| **Infant characteristics** |  |  |  |  |  |  |  |
| Female | 160/323 (49.5%) | 207/410 (50.5%) | 0.82 |  | 833/1655 (50.3%) | 1129/2319 (48.7%) | 0.32 |
| Mean birth weight (SD), kg | 3.00 (0.49) | 2.97 (0.51) | 0.38 |  | 3.10 (0.46) | 3.07 (0.53) | 0.24 |
| Birth weight <2500 g | 35/312 (11.2%) | 49/439 (11.2%) | 0.22 |  | 131/1574 (8.3%) | 195/2000 (9.8%) | 0.20 |
| Institutional delivery | 270/306 (88.2%) | 274/343 (79.9%) | 0.003 |  | 1401/1567 (89.4%) | 1807/2037 (88.7%) | 0.48 |
| Vaginal delivery | 290/314 (92.4%) | 319/345 (92.5%) | 0.96 |  | 1489/1606 (92.7%) | 1902/2058 (92.4%) | 0.74 |

^1^ Baseline variables presented for mothers who had live births. Maternal and household data collected about 2 weeks after consent (~14 weeks gestation); this gap created opportunity for loss to follow-up between consent and baseline, thus the number of mothers completing baseline visit is smaller than the number of mothers with live births. Baseline for infants was at birth. Values are %, unless noted. For variables where [n] is not stated, <3% of data are missing based on number of baseline visits completed.

^2^ CD4 count at baseline visit, or at 32 gestational week visit if no baseline result.

^3^ Documented antiretroviral therapy use during pregnancy

^4^ Documented co-trimoxazole prophylaxis use during pregnancy

^5^ Chasekwa B, Maluccio JA, Ntozini R, Moulton LH, Wu F, Smith LE, et al. Measuring wealth in rural communities: Lessons from the Sanitation, Hygiene, Infant Nutrition Efficacy (SHINE) trial. PLoS ONE. 2018; 13(6): e0199393.

SD: standard deviation; IQR: interquartile range; MUAC: Mid-upper arm circumference
