# Supplementary material for: Early child development in children who are HIV‐exposed uninfected compared to children who are HIV‐unexposed: observational sub‐study of a cluster‐randomized trial in rural Zimbabwe
Source: J Int AIDS Soc. 2020 May 9;23(5):e25456. doi: 10.1002/jia2.25456 (PMC7318086; doi:10.1002/jia2.25456)
Supplement: Supplementary file 2 — Appendix S1. Supplementary methods. [file JIA2-23-e25456-s002.pdf]

SUPPLEMENTARY APPENDIX

Early child development in HIV-exposed uninfected compared to Children who are HIV-unexposed: observational sub-study of a cluster-randomized trial in rural Zimbabwe

Robert Ntozini[1§], Jaya Chandna[1,2], Ceri Evans[1,3], Bernard Chasekwa[1], Florence D. Majo[1], Gwendoline Kandawasvika[4], Naume V. Tavengwa[1], Batsirai Mutasa[1], Kuda Mutasa[1], Lawrence H. Moulton[5], Jean H. Humphrey[1,5], Melissa J. Gladstone[2], Andrew J. Prendergast[1,3,5] for the SHINE Trial Team‡

§ **Corresponding author:** Robert Ntozini, 16 Lauchlan Avenue, Meyrick Park, Harare, Zimbabwe. Phone: 263 773 786 975 (rntozini@zvitambo.co.zw)

‡Membership of the SHINE Trial Team are listed in supplementary file (S2 Appendix)

1 Zvitambo Institute for Maternal and Child Health Research, Harare, Zimbabwe

2 University of Liverpool, Liverpool, UK

3 Blizard Institute, Queen Mary University of London, London, UK.

4 University of Zimbabwe, Harare, Zimbabwe

5 Department of International Health, Johns Hopkins Bloomberg School of Public Health, Baltimore MD, USA

Contents

Supplementary methods

a) Randomisation procedure p2-3

b) Changes in gestational age enrolment criteria p4

c) Further details of interventions p4-7

d) Validation and quality control of Early Child Development substudy p7

e) Definition of per protocol analysis p7

SUPPLEMENTARY METHODS

a) Randomisation procedure

A highly constrained randomisation technique was used to allocate clusters (stratified by district) to treatments. We randomly selected 1000 allocations from among 5000 computer-generated allocations that balanced the 4 treatment arms on 14 parameters without pre-specified bounds. From the 1000, we randomly selected 10 for a public randomisation ceremony; Figure A. Each randomisation scheme divided the randomisation units into 4 groups of approximately 53 units. Each scheme's corresponding colour-coded map was printed on a separate sheet and displayed at a public forum attended by all elected councillors from the study area, District and Provincial Administrators, and Ministry of Health and Child Care authorities. In their presence, 10 plastic balls (numbered 1-10) were placed in an opaque sack. A community representative selected one ball from the sack, thereby identifying which of the 10 numbered allocations would be used. Then, four balls (labelled A, B, C, and D) were placed in one sack, and four balls (labelled with the 4 treatment arms) were placed in a second sack. Representatives drew a ball from the first sack and a ball from the second sack, pairing a group of clusters with one of the four treatment arms, thereby mimicking a widely known World Cup draw procedure. This was repeated twice more to pair the next two groups of clusters with two more treatment arms. The remaining balls formed the final pairing. This second stage was included to provide an additional assurance of impartiality/randomness and a further opportunity for participation of the community leadership.

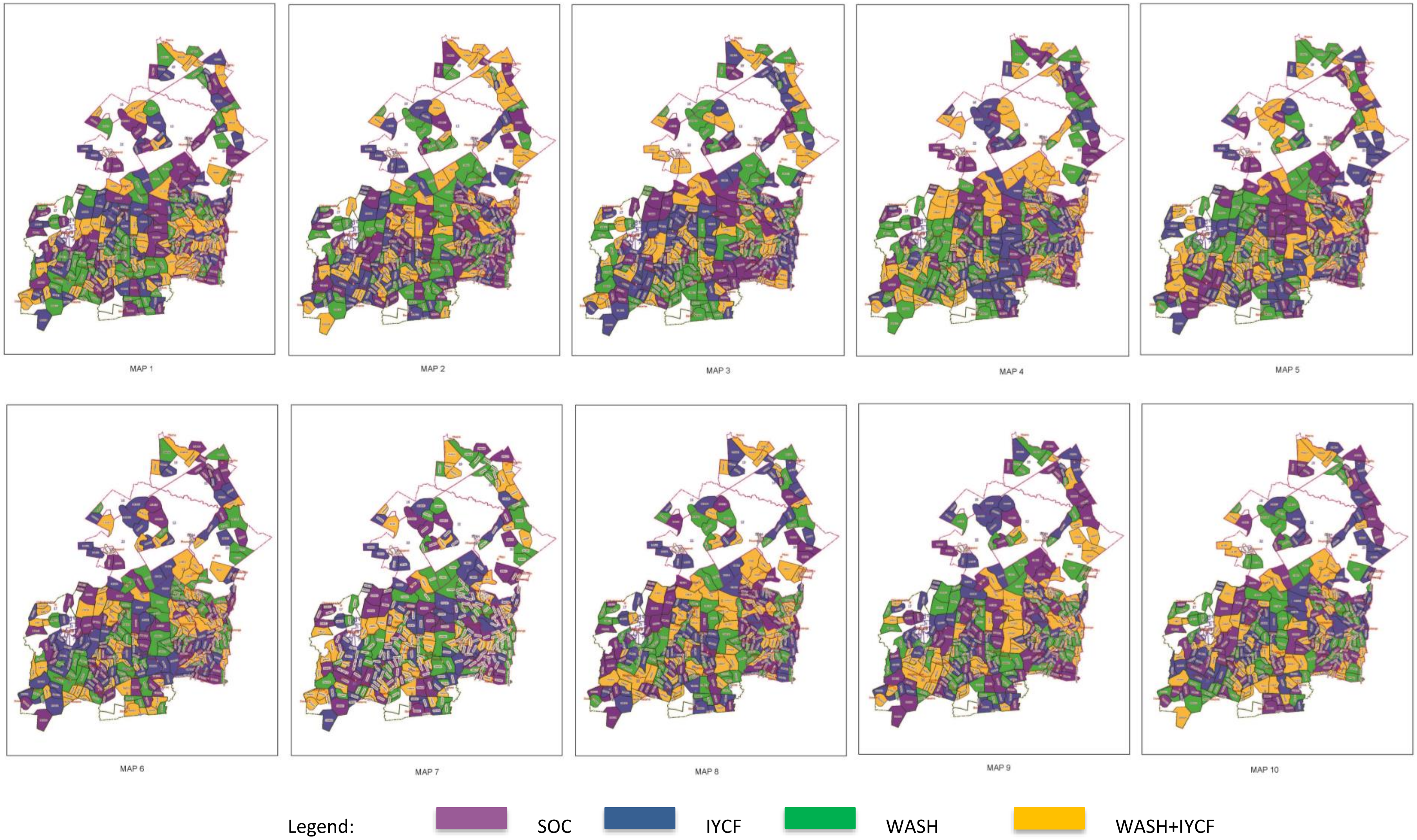

Figure A. Maps of ten SHINE randomization schemes

b) Changes in gestational age enrolment criteria

The goal at the start of the trial was to recruit women between 10-14 weeks gestation, so women more than 14 weeks gestation were excluded. However, this cut-off was liberalised three times over the life of the trial to maximise recruitment because many women were excluded due to pregnancy that was >14 gestational weeks at screening. The upper limit of gestational age was therefore increased to 18 weeks (August 22, 2013), 24 weeks (January 3, 2014), and any time prior to parturition (October 20, 2014), through trial protocol amendments.

c) Further details of interventions

Standard of Care (SOC) Intervention: Village Health Workers were trained through the Ministry of Health and Child Care curriculum, which instructs VHWs to visit pregnant women and infants frequently, although the precise content or purpose of each visit is not specified. Consequently the SHINE SOC intervention was designed to standardize the number of visits (3 antenatal and 12 postnatal visits) and the content of primary health care messages across treatment arms. Four of these visits promoted exclusive breastfeeding (EBF) from birth to 6 months using modules designed to overcome contextual barriers identified in formative work. Other SOC modules include prevention of mother-to-child HIV transmission (PMTCT), antenatal care, hospital-based delivery, family planning and immunizations.

WASH Intervention: Within 6 weeks of enrolment (~20 weeks gestation) into the WASH and WASH+IYCF arms of the trial, a Blair Ventilated Improved Pit (VIP) Latrine was constructed at the participant's household and two 'Tippy Tap' hand-washing stations (locally manufactured, and adapting the model piloted by the Kenya WASH Benefits trial) were installed near the latrine and kitchen; Figure B.

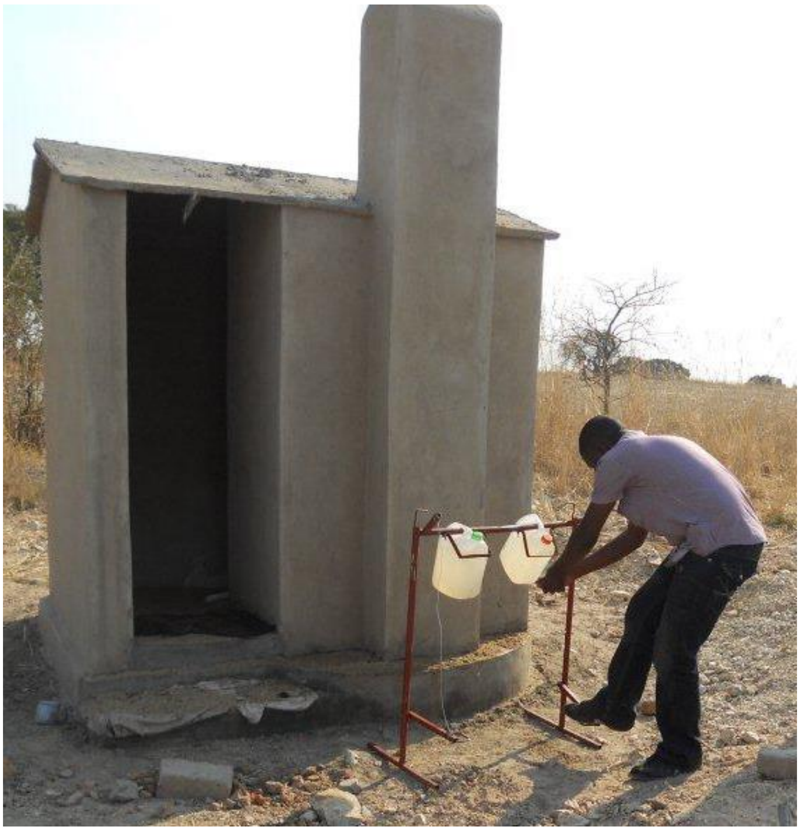

Figure B: Blair VIP latrine and Tippy Tap

WASH Modules 1 (delivered at 24 gestational weeks) and 2 (32 gestational weeks) promoted safe disposal of faeces, and hand-washing with soap after faecal contact and before food preparation and eating, respectively. Our intention was for the baby to be born into a household in which latrine use and household hand-washing behaviours were normalised and habitual. WASH Module 3 (protecting babies from faecal ingestion during exploratory play) was delivered when the baby was 2 months old; a washable 2.8m x 3.0m locally manufactured mat and plastic play yard

(North States, Minneapolis MN) were provided at 2 months and 6 months, respectively; Figure C.

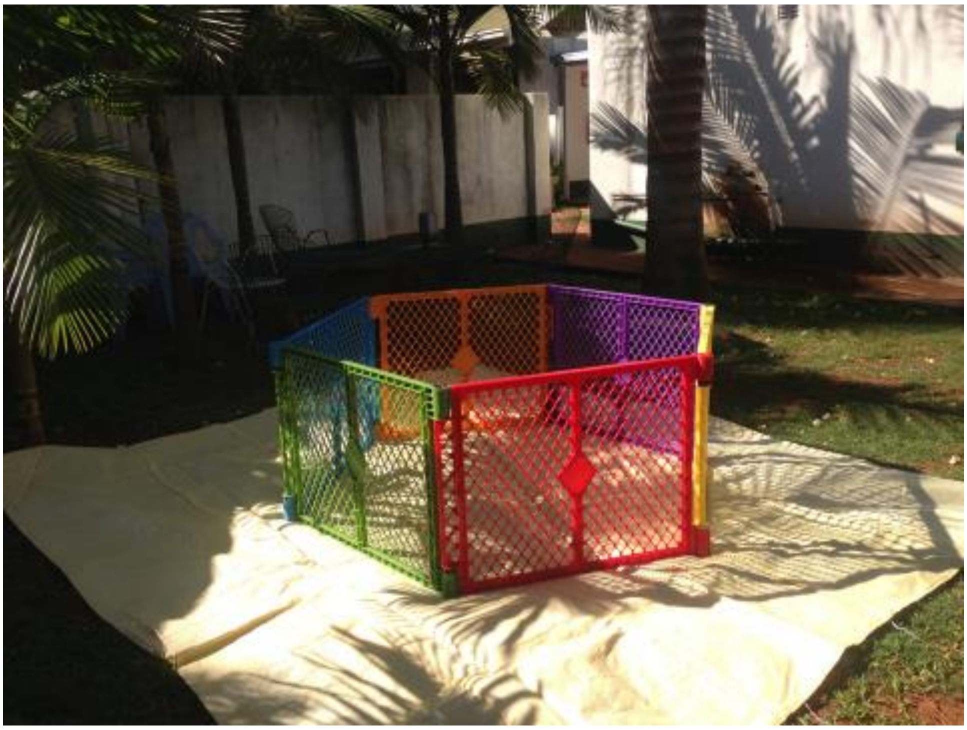

Figure C: SHINE mat and play yard

WASH Module 4 (treat all drinking water given to babies after 6 months of EBF) was delivered at 4 months of age, along with point-of-use chlorination (WaterGuard: a dilute sodium hypochlorite solution, manufactured locally by Nelspot). Liquid soap and Water Guard were regularly replenished from time of introduction (Module 2 and 5, respectively) until the infant was 18 months old. WASH Module 5, delivered at 5 months of age, stressed the importance of freshly preparing or fully reheating all foods fed to infants. A review module was delivered at 12 months.

IYCF Intervention: IYCF Module 1 (delivered at 5 months) linked good infant feeding to child growth, health, and intelligence. IYCF Module 2 (6 months) promoted feeding nutrient-dense food, including 20 g per day of the lipid-based nutrient supplement (LiNS) developed by the International Lipid-Based Nutrients Supplements Project, provided monthly when the baby was 6 to 18 months of age. Module 3 (7 months) was a participatory cooking demonstration in which any available household food was prepared and fed to the baby, stressing three messages from formative research: 1) an infant can eat any food that an adult eats; 2) food should be ground so that the infant can swallow and

digest it; 3) food that is locally available is important for the infant. Module 4 (8 months) promoted responsive feeding during illness, Module 5 (9 months) promoted diet diversity, and a review module was delivered at 12 months.

d) Validation and quality control of Early Child Development substudy

Supportive supervision was provided at least monthly for each nurse by the study psychologist (JC). Every 6 months, nurses conducted an ECD assessment which was observed and double-marked by JC. If percentage agreement was < 85%, the nurse was retrained and retested until achieving 85% before testing any further study children. Inter-class correlation between nurses for the same child was high: MDAT 0.88 (95% CI: 0.82 to 0.94); McArthur Bates 0.94 (95% CI: 0.90 to 0.96); A-not-B 0.85 (95% CI: 0.80 to 0.90) and self-control 0.80 (95% CI: 0.76 to 0.85). A 10% subsample of assessments were video-recorded and reviewed by the study psychologist (JC) and a paediatrician with advanced training in child neurodevelopment and Shona language proficiency (GK). Percentage agreement on recorded assessments was 93% for MDAT fine motor, 90% for MDAT language, 97% for A-not-B and 91% for the self-control task.

e) Definition of per protocol analysis

Secondary modified per protocol analyses were conducted with these restrictions:

WASH intervention

Higher Fidelity: received all 5 WASH modules and 12-month review

Lower Fidelity: received less than all 5 WASH modules and 12-month review

IYCF intervention

Higher Fidelity: received all 5 IYCF modules and 12-month review

Lower Fidelity: received less than all 5 IYCF modules and 12-month review
