# Supplementary material for: Early child development in children who are HIV‐exposed uninfected compared to children who are HIV‐unexposed: observational sub‐study of a cluster‐randomized trial in rural Zimbabwe
Source: J Int AIDS Soc. 2020 May 9;23(5):e25456. doi: 10.1002/jia2.25456 (PMC7318086; doi:10.1002/jia2.25456)
Supplement: Supplementary file 3 — Appendix S2. SHINE Team list. [file JIA2-23-e25456-s003.pdf]

Sanitation Hygiene Infant Nutrition Efficacy (SHINE) Trial Team

Members of the SHINE Trial team are listed at https://doi.org/10.1093/cid/civ844

First name	Initials	Lastname
Jean	H	Humphrey
Andrew	D	Jones
Amee		Manges
Goldberg		Mangwadu
John	A	Maluccio
Mduduzi	NN	Mbuya
Lawrence	H	Moulton
Robert		Ntozini
Andrew	J	Prendergast
Rebecca	J	Stoltzfus
James	M	Tielsch
Cynthia		Chasokela
Ancikaria		Chigumira
William		Heylar
Preston		Hwena
George		Kembo
Florence	D	Majo
Batsirai		Mutasa
Kuda		Mutasa
Philippa		Rambanepasi
Virginia		Sauramba
Naume	V	Tavengwa
Franne	Van Der	Keilen
Chipo		Zambezi
Dzivaidzo		Chidhanguro
Dorcas		Chigodora
Joseph	F	Chipanga
Grace		Gerema
Tawanda		Magara
Mandava		Mandava
Tafadzwa		Mavhudzi
Clever		Mazhanga
Grace		Muzaradope
Marian	T	Mwapaura
Simon		Phiri
Alice		Tengende
Cynthia		Banda
Bernard		Chasekwa
Leah		Chidamba
Theodore		Chidawanyika
Elisha		Chikwindi
Lovemore	K	Chingaona
Courage	K	Chiorera
Adlight		Dandadzi
Margaret		Govha

 Members of the SHINE Trial team are listed at https://doi.org/10.1093/cid/civ844

Hlanai		Gumbo
Karen	T	Gwanzura
Sarudzai		Kasaru
Rachel		Makasi
Alois	M	Matsika
Diana		Maunze
Exevia		Mazarura
Eddington		Mpofu
Johnson		Mushonga
Tafadzwa	E	Mushore
Tracey		Muzira
Netsai		Nembaware
Sibongile		Nkiwane
Penias		Nyamwino
Sandra	D	Rukobo
Thompson		Runodamoto
Shepherd		Seremwe
Pururudzai		Simango
Joice		Tome
Blessing		Tsenesa
Umali		Amadu
Beauty		Bangira
Daniel		Chiveza
Priscilla		Hove
Horaiti	A	Jombe
Didymus		Kujenga
Lenin		Madhuyu
Prince	M	Makoni
Naume		Maramba
Betty		Maregere
Ellen		Marumani
Elisha		Masakadze
Phathisiwe		Mazula
Caroline		Munyanyi
Grace		Musanhu
Raymond	C	Mushanawani
Sibongile		Mutsando
Felicia		Nazare
Moses		Nyarambi
Wellington		Nzuda
Trylife		Sigauke
Monica		Solomon
Tendai		Tavengwa
Farisai		Biri
Misheck		Chafanza
Cloud		Chaitezvi
Tsundukani		Chauke
Collen		Chidzomba
Tawanda		Dadirai
Clemence		Fundira

Athanasios	C	Gambiza
Tatenda		Godzongere
Maria		Kuona
Tariro		Mafuratidze
Idah		Mapurisa
Tsitsi		Mashedze
Nokuthula		Moyo
Charles		Musariri
Matambudzo		Mushambadope
Tawanda	R	Mutsonziwa
Augustine		Muzondo
Rudo		Mwareka
Juleika		Nyamupfukudza
Baven		Saidi
Tambudzai		Sakuhwehwe
Gerald		Sikalima
Jenneth		Tembe
Tapiwanashe	E	Chekera
Owen		Chihombe
Muchaneta		Chikombingo
Tichaona		Chirinda
Admire		Chivizhe
Ratidzai		Hove
Rudo		Kufa
Tatenda	F	Machikopa
Wilbert		Mandaza
Liberty		Mandongwe
Farirai		Manhiyo
Emmanuel		Manyaga
Peter		Mapuranga
Farai	S	Matimba
Patience		Matonhodze
Sarah		Mhuri
Joice		Mike
Bekezela		Ncube
Walter	TS	Nderecha
Munyaradzi		Noah
Charles		Nyamadzawo
Jonathan		Penda
Asinje		Saidi
Sarudzai		Shonhayi
Clemence		Simon
Monica		Tichagwa
Rachael		Chamakono
Annie		Chauke
Andrew	F	Gatsi
Blessing		Hwena
Hillary		Jawi
Benjamin		Kaisa
Sithembile		Kamutanho

Tapiwa		Kaswa
Paradhi		Kayeruza
Juliet		Lunga
Nomatter		Magogo
Daniel		Manyeruke
Patricia		Mazani
Fungai		Mhuriyengwe
Farisai		Mlambo
Stephen		Moyo
Tawanda		Mpofu
Mishelle		Mugava
Yvonne		Mukungwa
Fungai		Muroyiwa
Eddington		Mushonga
Selestino		Nyekete
Tendai		Rinashe
Kundai		Sibanda
Milton		Chemhuru
Jeffrey		Chikunya
Vimbai	F	Chikwavaire
Charity		Chikwiriro
Anderson		Chimusoro
Jotam		Chinyama
Gerald		Gwinji
Nokuthula		Hoko-Sibanda
Rutendo		Kandawasvika
Tendai		Madzimure
Brian		Maponga
Antonella		Mapuranga
Joana		Marembo
Luckmore		Matsunge
Simbarashe		Maunga
Mary		Muchekeza
Monica		Muti
Marvin		Nyamana
Efa		Azhuda
Urayai		Bhoroma
Ailleen		Biriyadi
Elizabeth		Chafota
Angelline		Chakwizira
Agness		Chamhamiwa
Tavengwa		Champion
Stella		Chazuza
Beauty		Chikwira
Chengeto		Chingozho
Abigail		Chitabwa
Annamary		Dhurumba
Albert		Furidzirai
Andrew		Gandanga
Chipo		Gukuta

Beauty	Macheche
Bongani	Marihwi
Barbara	Masike
Eunice	Mutangandura
Beatrice	Mutodza
Angeline	Mutsindikwa
Alice	Mwale
Rebecca	Ndhlovu
Norah	Nduna
Cathrine	Nyamandi
Elias	Ruvata
Babra	Sithole
Rofina	Urayai
Bigboy	Vengesa
Micheal	Zorounye
Memory	Bamule
Michael	Bande
Kumbirai	Chahuruva
Lilian	Chidumba
Zvisinei	Chigove
Kefas	Chiguri
Susan	Chikuni
Ruvarashe	Chikwanda
Tarisai	Chimbi
Micheal	Chingozho
Olinia	Chinhamo
Regina	Chinokuramba
Chiratidzo	Chinyoka
Xaviour	Chipenzi
Raviro	Chipute
Godfrey	Chiribhani
Mary	Chitsinga
Charles	Chiwanga
Anamaria	Chiza
Faith	Chombe
Memory	Denhere
Ephania	Dhamba
Miriam	Dhamba
Joyas	Dube
Florence	Dzimbanhete
Godfrey	Dzingai
Sikhutele	Fusira
Major	Gonese
Johnson	Gota
Kresencia	Gumure
Phinias	Gwaidza
Margret	Gwangwava
Winnet	Gwara
Melania	Gwauya
Maidei	Gwiba

Joyce	Hamauswa
Sarah	Hlasera
Eustina	Hlukani
Joseph	Hotera
Lovemore	Jakwa
Gilbert	Jangara
Micheal	Janyure
Christopher	Jari
Duvai	Juru
Tabeth	Kapuma
Paschalina	Konzai
Moly	Mabhodha
Susan	Maburutse
Chipo	Macheka
Tawanda	Machigaya
Florence	Machingauta
Eucaria	Machokoto
Evelyn	Madhumba
Learnard	Madziise
Clipps	Madziva
Mavis	Madzivire
Mistake	Mafukise
Marceline	Maganga
Senzeni	Maganga
Emmanuel	Mageja
Miriam	Mahanya
Evelyn	Mahaso
Sanelisiwe	Mahleka
Pauline	Makanhiwa
Mavis	Makarudze
Constant	Makeche
Nickson	Makopa
Ranganai	Makumbe
Mascline	Mandire
Eunice	Mandiyanike
Eunice	Mangena
Farai	Mangiro
Alice	Mangwadu
Tambudzai	Mangwengwe
Juliet	Manhidza
Farai	Manhovo
Irene	Manono
Shylet	Mapako
Evangelista	Mapfumo
Timothy	Mapfumo
Jane	Mapuka
Douglas	Masama
Getrude	Masenge
Margreth	Mashasha
Veronica	Mashivire

Moses	Matunhu
Pazvichaenda	Mavhoro
Godfrey	Mawuka
Ireen	Mazango
Netsai	Mazhata
David	Mazuva
Mary	Mazuva
Filomina	Mbinda
John	Mborera
Upenyu	Mfiri
Florence	Mhandu
Chrispen	Mhike
Tambudzai	Mhike
Artwell	Mhuka
Judith	Midzi
Siqondeni	Moyo
Michael	Mpundu
Nicholas	Msekiwa
Dominic	Msindo
Choice	Mtisi
Gladys	Muchemwa
Nyadziso	Mujere
Ellison	Mukaro
Kilvera	Muketiwa
Silvia	Mungoi
Esline	Munzava
Rosewita	Muoki
Harugumi	Mupura
Evelyn	Murerwa
Clarieta	Murisi
Letwin	Muroyiwa
Musara	Muruvi
Nelson	Musemwa
Christina	Mushure
Judith	Mutero
Philipa	Mutero
Patrick	Mutumbu
Cleopatra	Mutya
Lucia	Muzanango
Martin	Muzembi
Dorcus	Muzungunye
Valeliah	Mwazha
Thembeni	Ncube
Takunda	Ndava
Nomvuyo	Ndlovu
Pauline	Nehowa
Dorothy	Ngara
Leonard	Nguruve
Petronella	Nhigo
Samukeliso	Nkiwane

Luckson	Nyanyai
Judith	Nzombe
Evelyn	Office
Beatrice	Paul
Shambadzirai	Pavari
Sylvia	Ranganai
Stella	Ratisai
Martha	Rugara
Peter	Rusere
Joyce	Sakala
Prosper	Sango
Sibancengani	Shava
Margaret	Shekede
Cornellious	Shizha
Tedla	Sibanda
Neria	Tapambwa
John	Tembo
Netsai	Tinago
Violet	Tinago
Theresa	Toindepi
John	Tovigepi
Modesta	Tuhwe
Kundai	Tumbo
Tinashe	Zaranyika
Tongai	Zaru
Kamurayi	Zimidzi
Matilda	Zindo
Maria	Zindonda
Nyaradzai	Zinhumwe
Loveness	Zishiri
Emerly	Ziyambi
James	Zvinowanda
Ekenia	Bepete
Christine	Chiwira
Naume	Chuma
Abiegirl	Fari
Samson	Gavi
Violet	Gunha
Fadzai	Hakunandava
Constance	Huku
Given	Hungwe
Grace	Maduke
Elliot	Manyewe
Tecla	Mapfumo
Innocent	Marufu
Chenesai	Mashiri
Shellie	Mazenge
Euphrasia	Mbinda
Abigail	Mhuri
Charity	Muguti

Lucy	Munemo
Loveness	Musindo
Laina	Ngada
Dambudzo	Nyembe
Rachel	Taruvinga
Emma	Tobaiwa
Selina	Banda
Jesca	Chaipa
Patricia	Chakaza
Macdonald	Chandigere
Annie	Changunduma
Chenesai	Chibi
Otilia	Chidyagwai
Elika	Chidza
Nora	Chigatse
Lennard	Chikoto
Vongai	Chingware
Jaison	Chinhamo
Marko	Chinhoro
Answer	Chiripamberi
Esther	Chitavati
Rita	Chitiga
Nancy	Chivanga
Tracy	Chivese
Flora	Chizema
Sinikiwe	Dera
Annacolleta	Dhliwayo
Pauline	Dhononga
Ennia	Dimingo
Memory	Dziyani
Tecla	Fambi
Lylian	Gambagamba
Sikangela	Gandiyari
Charity	Gomo
Sarah	Gore
Jullin	Gundani
Rosemary	Gundani
Lazarus	Gwarima
Cathrine	Gwaringa
Samuel	Gwenya
Rebecca	Hamilton
Agnes	Hlabano
Ennie	Hofisi
Florence	Hofisi
Stanley	Hungwe
Sharai	Hwacha
Aquiiline	Hwara
Ruth	Jogwe
Atanus	Kanikani
Lydia	Kuchicha

Mitshel	Kutsira
Kumbulani	Kuziyamisa
Mercy	Kuziyamisa
Benjamin	Kwangware
Portia	Lozani
Joseph	Mabuto
Vimbai	Mabuto
Loveness	Mabvurwa
Rebecca	Machacha
Cresenzia	Machaya
Roswitha	Madembo
Susan	Madya
Sheneterai	Madzingira
Lloyd	Mafa
Fungai	Mafuta
Jane	Mafuta
Alfred	Mahara
Sarudzai	Mahonye
Admire	Maisva
Admire	Makara
Margreth	Makover
Ennie	Mambongo
Murenga	Mambure
Edith	Mandizvidza
Gladys	Mangena
Elliot	Manjengwa
Julius	Manomano
Maria	Mapfumo
Alice	Mapfurire
Letwin	Maphosa
Jester	Mapundo
Dorcas	Mare
Farai	Marecha
Selina	Marecha
Christine	Mashiri
Medina	Masiya
Thembinkosi	Masuku
Priviledge	Masvimbo
Saliwe	Matambo
Getrude	Matarise
Loveness	Matinanga
John	Matizanadzo
Margret	Maunganidze
Belinda	Mawere
Chipiwa	Mawire
Yulliana	Mazvanya
Maudy	Mbasera
Magret	Mbono
Cynthia	Mhakayakora
Nompumelelo	Mhlanga

Bester	Mhosva
Nomuhle	Moyo
Over	Moyo
Robert	Moyo
Charity	Mpakami
Rudo	Mpedzisi
Elizabeth	Mpofu
Estery	Mpofu
Mavis	Mtetwa
Juliet	Muchakachi
Tsitsi	Mudadada
Kudakwashe	Mudzingwa
Mejury	Mugwira
Tarsisio	Mukarati
Anna	Munana
Juliet	Munazo
Otilia	Munyeki
Patience	Mupfeka
Gashirai	Murangandi
Maria	Muranganwa
Josphine	Murenjekwa
Nothando	Muringo
Tichafara	Mushaninga
Florence	Mutaja
Dorah	Mutanha
Peregia	Mutemeri
Beauty	Mutero
Edina	Muteya
Sophia	Muvembi
Tandiwe	Muzenda
Agnes	Mwenjota
Sithembisiwe	Ncube
Tendai	Ndabambi
Nomsa	Ndava
Elija	Ndlovu
Eveln	Nene
Enniah	Ngazimbi
Atalia	Ngwalati
Tafirenyika	Nyama
Agnes	Nzembe
Eunica	Pabwaungana
Sekai	Phiri
Ruwiza	Pukuta
Melody	Rambanapasi
Tambudzai	Rera
Violet	Samanga
Sinanzeni	Shirichena
Chipiwa	Shoko
More	Shonhe
Cathrine	Shuro

Members of the SHINE Trial team are listed at https://doi.org/10.1093/cid/civ844

Juliah		Sibanda
Edna		Sibangani
Nikisi		Sibangani
Norman		Sibindi
Mercy		Sitotombe
Pearson		Siwawa
Magret		Tagwirei
Pretty		Taruvinga
Antony		Tavagwisa
Esther		Tete
Yeukai		Tete
Elliot		Thandiwe
Amonilla		Tibugari
Stella		Timothy
Rumbidzai		Tongogara
Lancy		Tshuma
Mirirayi		Tsikira
Constance		Tumba
Rumbidzayi		Watinaye
Ethel		Zhiradzango
Esther		Zimunya
Leanmary		Zinengwa
Magret		Ziupfu
Job		Ziyambe
James	A	Church
Amy		Desai
Dadirai		Fundira
Ethan		Gough
Rukundo	A	Kambarami
Cynthia	R	Matare
Thokozile	R	Malaba
Tatenda		Mupfudze
Francis		Ngure
Laura	E	Smith
Val		Curtis
Katherine	L	Dickin
Jean-Pierre		Habicht
Collen		Masimirembwa
Peter		Morgan
Gretel	H	Pelto
Corinne		Sheffner-Rogers
Roslyn		Thelingwani
Paul		Turner
Lindiwe		Zungu
Tariro		Makadzange
Hilda	A	Mujuru
Chandiwana		Nyachowe
Rugare		Chakadai
Gabriel		Chanyau
Mary	G	Makamure

Members of the SHINE Trial team are listed at https://doi.org/10.1093/cid/civ844

Humphrey	Chiwariro
Tambudzai	Mtetwa
Jeffrey	Chikunya
Lisbern	Maguwu
Simon	Nyadundu
Tshebukani	Moyo
Beauty	Chayima
Lucy	Mvindi
Pauline	Rwenhamo
Shamiso	Muzvarwandoga
Rumbidzai	Chimukangara
Handrea	Njovo
Talent	Makoni

Members of the SHINE Trial team are listed at https://doi.org/10.1093/cid/civ844
